# Inside out: Bone marrow adipose tissue as a source of circulating adiponectin

**DOI:** 10.1080/21623945.2016.1149269

**Published:** 2016-03-22

**Authors:** Erica L. Scheller, Aaron A. Burr, Ormond A. MacDougald, William P. Cawthorn

**Affiliations:** a Division of Bone and Mineral Diseases, Department of Internal Medicine, Washington University, Saint Louis, MO, USA; b Department of Molecular & Integrative Physiology; University of Michigan Medical School; Ann Arbor, MI, USA; c Department of Internal Medicine; University of Michigan Medical School; Ann Arbor, MI, USA; d Program in Cellular and Molecular Biology, University of Michigan Medical School, Ann Arbor, MI, USA; e University/BHF Center for Cardiovascular Science, The Queen's Medical Research Institute, University of Edinburgh, Edinburgh, UK

**Keywords:** adiponectin, anorexia nervosa, bone marrow adipose tissue, caloric restriction, lipodystrophy, obesity, white adipose tissue

## Abstract

The adipocyte-derived hormone adiponectin mediates beneficial cardiometabolic effects, and hypoadiponectinemia is a biomarker for increased metabolic and cardiovascular risk. Indeed, circulating adiponectin decreases in obesity and insulin-resistance, likely because of impaired production from white adipose tissue (WAT). Conversely, lean states such as caloric restriction (CR) are characterized by hyperadiponectinemia, even without increased adiponectin production from WAT. The reasons underlying this paradox have remained elusive, but our recent research suggests that CR-associated hyperadiponectinemia derives from an unexpected source: bone marrow adipose tissue (MAT). Herein, we elaborate on this surprising discovery, including further discussion of potential mechanisms influencing adiponectin production from MAT; additional evidence both for and against our conclusions; and observations suggesting that the relationship between MAT and adiponectin might extend beyond CR. While many questions remain, the burgeoning study of MAT promises to reveal further key insights into MAT biology, both as a source of adiponectin and beyond.

## Introduction

It is now well established that white adipose tissue (WAT) is a major endocrine organ, with white adipocytes secreting numerous hormones, cytokines, lipids, and other molecules to exert diverse local and systemic effects.^1^ Notable among these diverse endocrine factors, known as adipokines, is the hormone adiponectin. First discovered in 1995, adiponectin is the most abundant adipokine in the circulation, where it exists in distinct multimeric forms including low-molecular-weight (LMW) trimers, middle-molecular-weight (MMW) hexamers, and high-molecular-weight (HMW) complexes including dodecamers and even larger multimers. Over the past 20 years adiponectin has become established as a major topic of biomedical research that, at the time of writing (January 2016), has been featured in over 15,000 published studies. Such interest reflects the diverse biological actions of adiponectin, as well as its utility as a biomarker for increased risk of clinical conditions including insulin resistance, cardiovascular diseases, bone loss, and certain cancers.^2^

While such extensive study has yielded great insights, many questions remain unsolved. One notable question concerns the so-called adiponectin paradox: despite being produced exclusively by adipose tissue, circulating adiponectin levels decrease in conditions of excess adiposity (i.e. obesity) but are elevated markedly in conditions of extreme leanness, such as during caloric restriction (CR) in animals and in human subjects with anorexia nervosa (AN). In obese, insulin-resistant states, hypoadiponectinemia likely results from decreased expression and secretion of adiponectin transcripts and protein in WAT.^3^ In contrast, why circulating adiponectin increases in CR and AN has remained poorly understood.

In research published last year we revealed that, during CR, increased circulating adiponectin comes from a previously unrecognized source: bone marrow adipose tissue (MAT).^4^ Herein we elaborate on these findings, including additional lines of evidence that further support our conclusions, and other data and clinical observations suggesting that the relationship between MAT and circulating adiponectin, both in CR and beyond, may be more complex. While there is much evidence to consider, many key questions remain to be addressed.

## Increased adiponectin during CR: WAT's going on?

Four years after adiponectin was identified, Arita and colleagues made the surprising discovery that, despite being produced by adipocytes, circulating adiponectin concentrations correlate inversely with adiposity.^5^ Thus, states of obesity and insulin resistance are characterized by hypoadiponectinemia. It is now well established that this results not from increased adiponectin clearance,^6^ but from impaired adiponectin production from WAT.^3^ Indeed, adiponectin has been studied extensively in the context of obesity and insulin resistance. In contrast, research into hyperadiponectinemia during CR has been more limited. The first reports of this phenomenon emerged in 2003, beginning with the observation that circulating adiponectin is significantly increased in humans with AN.^7^ This finding coincided with a study from Phil Scherer's group, which revealed that, in lean mice, chronic CR leads to hyperadiponectinemia.^8^ These initial reports have since been followed by numerous additional studies that, with some exceptions, further demonstrate that circulating adiponectin levels are increased in subjects with AN or during CR in lean animals or humans (Table 1). However, while this phenomenon is now well established, the underlying mechanisms are not so clear. Hyperadiponectinemia can result from increased adiponectin production and/or decreased clearance from the circulation, but few studies of AN or CR have investigated these readouts (Table 1). Indeed, only one study of AN patients has measured adiponectin transcript expression in WAT, finding this to be decreased with AN.^9^ Surprisingly, no studies have analyzed the impact of AN on adiponectin half-life in the circulation. Whether AN alters expression or secretion of adiponectin protein in WAT also remains untested, perhaps owing to the difficulty of obtaining sufficient WAT from these extremely lean subjects. Other studies of CR in animals or humans have been similarly limited (Table 1), with none assessing secretion and only one analyzing the half-life of adiponectin, which was unaltered by CR.^10^ Unlike for AN, animal studies of CR have more thoroughly investigated adiponectin expression in WAT; however, the results have been mixed. Thus, several reports suggest that adiponectin transcripts are increased in WAT with CR,^10-17^ with three of these studies also finding increased expression of adiponectin protein.^10-12^ However, Wiesenborn et al. found that, despite increased transcript expression, adiponectin protein in WAT was *decreased* with CR.^16^ In contrast, studies from 6 other groups, including the Scherer lab and ourselves, find that CR is associated with unaltered or decreased expression of adiponectin mRNA in WAT.^4,8,18-21^ In our study, adiponectin protein expression was also unaltered.^4^

**Table 1.** Studies investigating the relationship between AN or CR, circulating adiponectin, WAT, and MAT.

		Effect of CR			
Study type	Readout	Increased	Unaltered	Decreased	Not analysed
AN (humans)	*Circulating adiponectin*	^4,7,9,97-103^	^104-106^	^107,108^	^109-113^
	*Adiponectin mRNA expression in WAT*			^9^	^4,7,97-113^
	*Adiponectin protein expression in WAT*				^4,7,97-113^
	*Adiponectin secretion from WAT*				^4,7,97-113^
	*Adiponectin half-life in circulation*				^4,7,97-113^
	*MAT volume / Bone marrow adiposity*	^4,109-113^			^7,9,97-108^
	Adiponectin *mRNA expression in MAT / bone*				^4,7,97-113^
CR (lean animals)	*Circulating adiponectin*	^4,8,10,12-17,114-120^	^19,21,121-123^	^21^	^11,18,20^
	*Adiponectin mRNA expression in WAT*	^10-17^	^4,8,18,19,21^	^20,21^	^114-123^
	*Adiponectin protein expression in WAT*	^10-12^	^4^	^16^	^8,13-15,17-21,114-123^
	*Adiponectin secretion from WAT*				^4,8,10-21,114-123^
	*Adiponectin half-life in circulation*		^10^		^4,8,11-21,114-123^
	*MAT volume*	^4^			^8,10-21,114-123^
	*Adiponectin mRNA expression in MAT / bone*	^4^			^8,10-21,114-123^
CR (lean humans)	*Circulating adiponectin*	^124,125^	^126^	^127,128^	
	*Adiponectin mRNA expression in WAT*				^124-128^
	*Adiponectin protein expression in WAT*				^124-128^
	*Adiponectin secretion from WAT*				^124-128^
	*Adiponectin half-life in circulation*				^124-128^
	*MAT volume*				^124-128^
	*Adiponectin mRNA expression in MAT / bone*				^124-128^

**Table 2.** **Impact of human lipodystrophies on WAT, MAT, and circulating adiponectin**. Data for MAT and WAT phenotypes are based on^63,133-138^ and/or studies discussed in a previous review.^81^ Data for adiponectin are based on the references indicated in the right-most column. Circulating adiponectin concentrations (mg/L) from each cohort are shown as (**median**; range). ^a^CGL1 and CGL2 are grouped together in this row because the study by Haque *et al.*^129^ did not distinguish between these two classes of CGL. ^b^In FPLD, visceral WAT content is normal in some subjects^134^ but increased in others,^138^ while in AGL the extent of WAT loss depends on the subtype of AGL^136^ – in most subjects, loss of scWAT is severe, while visceral (intra-abdominal) WAT content can be absent, normal, or increased. ^c^Data for CGL3 are from only a single subject, and therefore the reproducibility of this observation remains unknown; however, we recently confirmed maintenance of MAT in *Cav1-*knockout mice.^49^

		Phenotype				
	Class of lipodystrophy (*mutated gene*)	Visceral WAT	Subcutaneous WAT	MAT	Circulating adiponectin	Refs.
*MAT present, adiponectin normal (or small decrease)*	APL	Partial loss	Partial loss	Present	Normal (**7.9;** 3.1–13.3)	^129^
	FPLD (*LMNA)*	Variable^b^	Partial loss	Present	Normal (**6.4**; 1.9–23.2)	^129^
					Decreased (**3.9**; 1.4–15.2)	^130^
*MAT and adiponectin decreased*	HIV-associated	Increased	Partial loss	Partial loss	Decreased (**2.1**; 0.2–12.4)	^130^
	CGL4 (*PTRF)*	Absent	Absent	Partial loss	Large decrease (**0.5**; <0.4–2.3)	^131,132^
	CGL1 (*AGPAT2)*	Absent	Absent	Absent	Large decrease (**0.5**; <0.1–1.4)	^130^
	CGL2 (*BSCL2)*	Absent	Absent	Absent	Decreased (**3.3**; 0.7–23.7)	^130^
	CGL1/2 (*AGPAT2 or BSCL2)*^a^	Absent	Absent	Absent	Large decrease (**1.5**; 0.4–7.5)	^129^
*MAT present, adiponectin decreased*	AGL	Variable^b^	Large decrease^b^	Present	Decreased (**3.2**; 0.6–7.7)	^129^
	CGL3 (*CAV1)*	Absent	Absent	Present	Large decrease^c^ (**0.1**; N/A)	^133^
*Healthy controls*		Present	Present	Present	Normal (**7.8**; 1.5–29.4)	^130^

Together, these observations paint a murky picture: although CR can lead to increased adiponectin mRNA and protein expression in WAT, it can also promote hyperadiponectinemia even when the expression of adiponectin in WAT is unaltered or even decreased. Moreover, a recent study found that, in humans, changes in circulating adiponectin during CR are not associated with changes in adiponectin secretion from WAT.^22^ Adiponectin transcript expression in WAT is also unrelated to the changes in circulating adiponectin that occur in response to insulin or thiazolidinedione (TZD) treatment.^14,23^ Collectively, these findings question the assumption that WAT is the key source of increased circulating adiponectin during CR, and perhaps in other contexts. But if not WAT, then what?

## Digging deeper: bone marrow adipose tissue

**Figure 1.**
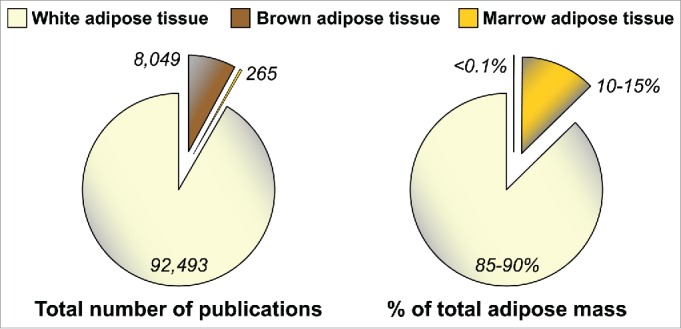
**MAT is under-studied, despite being a major adipose depot**. Numbers of publications featuring WAT, BAT or MAT were determined by searching the PubMed database in October 2015 with the following terms: WAT, “adipose tissue” OR “adipocyte” NOT “brown adipose tissue” NOT “brown adipocyte;” BAT, brown adipose tissue OR brown adipocyte; MAT, “marrow adipose tissue” OR “marrow adipocyte” OR “yellow marrow” OR “yellow bone marrow.” Values for WAT, BAT or MAT as percentage of total adipose mass in lean, healthy humans, are based on previous publications.^4,96^

One limitation to these studies is that their focus has been largely limited to WAT. This, perhaps, is unsurprising: WAT has been featured in over 90,000 published studies (Fig. 1), reflecting the widespread interest in this tissue. Brown adipose tissue (BAT), a distinct type of fat specialized for mediating adaptive thermogenesis, has also been subject to extensive research (Fig. 1), partly because of its promise as a therapeutic target for obesity and associated metabolic diseases.^24^ In contrast, modern biomedical research has largely ignored MAT (Fig. 1). This is surprising, because adipocytes in bone marrow (BM) were identified over a century ago and MAT accounts for up to 70% of BM volume in healthy humans.^25^ Moreover, our recent research suggests that healthy adults have over 2 kg of MAT, representing more than 10% of total adipose mass (Fig. 1).^4^ MAT further increases in diverse clinical conditions, including during CR in animals and in human subjects with AN.^26^ This is particularly striking given that such catabolic states typically feature WAT loss. *Thus, both MAT and circulating adiponectin are elevated during CR and AN*. This observation was the foundation for our hypothesis that MAT contributes to hyperadiponectinemia during CR; however, given the relatively limited knowledge of MAT biology, our efforts to address this hypothesis had to begin by focusing on some very basic questions.

## Production of adiponectin by MAT

### Adiponectin expression in MAT vs WAT

**Figure 2.**
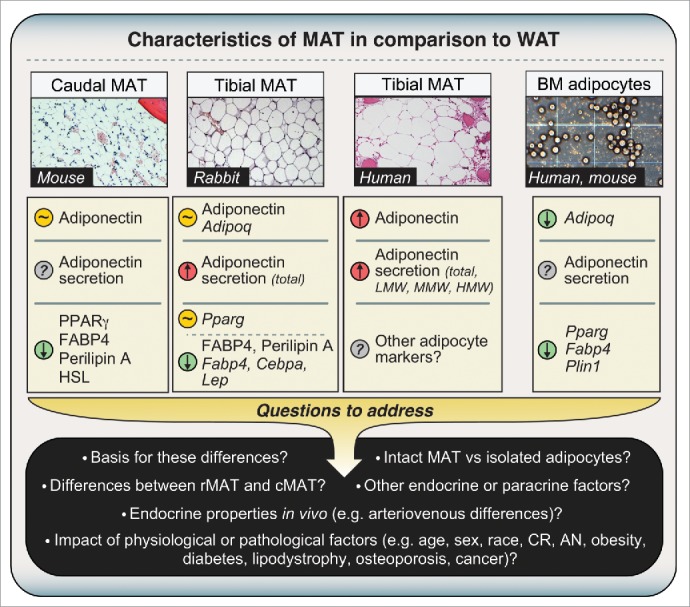
**Characteristics of MAT in comparison to WAT**. Expression or secretion of each factor, relative to WAT, is indicated as follows: greater than WAT, red circle with upward arrow; lower than WAT, green circle with downward arrow; similar to WAT, amber circle with ‘∼’; unknown, gray circle with ‘?’. Where differences refer to mRNA expression, official transcript names are used as follows: *Adipoq*, adiponectin; *Pparg*, PPARγ; *Fabp4*, FABP4; *Cebpa*, C/EBPα; *Lep*, leptin; *Plin1*, Perilipin A. All other differences refer to expression or secretion of proteins. Micrographs for caudal and tibial MAT are H&E-stained sections from mice, rabbits or humans, as indicated. The micrograph of isolated adipocytes is a phase-contrast image of adipocytes from femoral MAT, post-isolation, and is presented for schematic purposes only. Characteristics of caudal and tibial MAT are based on our previously published observations.^4^ Characteristics of isolated BM adipocytes are based studies of MAT obtained from tibiae/femurs of mice or the iliac crest of humans.^36,46^ These observations demonstrate that MAT expresses and secretes adiponectin, but many questions remain to be addressed. Abbreviations and other details are given in the main text.

The first question was whether MAT even expresses adiponectin, and how this compares to expression in WAT. Adiponectin transcript and protein expression has been reported in whole BM of long bones of mice^27-31^ and in cultured BM adipocytes isolated from human femurs,^32^ with mRNA expression also noted in adipocytes differentiated *in vitro* from mouse or human BM stromal cells.^33,34^ Although such cultured adipocytes may not accurately mimic BM adipocyte characteristics *in vivo*, at least one report demonstrates that adipocytes within intact human BM express adiponectin mRNA and protein.^35^ However, none of these previous studies analyzed intact MAT and WAT samples. Therefore, we began by characterizing MAT and WAT obtained from mice, rabbits, and humans.^4^ In mice, techniques for isolation of intact MAT are yet to be perfected; hence, to study murine MAT we exploited the fact that BM of caudal (tail) vertebrae is essentially all MAT, with very little red, haematopoietic marrow (Fig. 2). We found that such caudal vertebrae express adiponectin protein at levels similar to those in inguinal WAT (iWAT), gonadal WAT (gWAT), and perirenal WAT (pWAT). In contrast, caudal vertebrae have far lower expression of other typical adipocyte markers, including peroxisome proliferator-activated receptor-γ (PPARγ), fatty-acid-binding protein 4 (FABP4), hormone-sensitive lipase (HSL), and perilipin A (Fig. 2).^4^ Another study also finds that, at the mRNA level, adipocytes isolated from mouse BM express PPARγ (*Pparg*), FABP4 *(Fabp4*), and perilipin A (*Plin1*) at lower levels than adipocytes from WAT (Fig. 2).^36^ This suggests that, in comparison to WAT, MAT expresses adiponectin transcripts and protein at higher levels than other adipocyte markers. Consistent with this, we found that, in rabbits, tibial MAT expresses adiponectin transcripts and protein at similar levels to iWAT, pWAT, and gWAT, while the expression of other typical adipocyte transcripts (e.g. *Cebpa, Fabp4*) and proteins (Perilipin A, FABP4) is lower than in each of these WAT depots (Fig. 2).^4^

This disparity between adiponectin expression and that of other adipocyte markers is striking, especially considering that the transcription factors PPARγ and CCAAT/enhancer-binding protein-α (C/EBPα) positively regulate adiponectin expression and secretion.^37-42^ Indeed, *Fabp4* is a key transcriptional target of PPARγ; hence, decreased *Fabp4* expression in MAT suggests diminished PPARγ activity. Why then would adiponectin be similarly expressed between MAT and WAT? Although the reason is unclear, other studies show that adiponectin expression can be uncoupled from that of other adipocyte genes in certain contexts.^39^ One possibility is that the different environments of MAT and WAT are responsible for their distinct expression of adipocyte markers. For example, BM adipocytes exist in close proximity to osteoblasts, and the osteoblast-secreted factor osteocalcin stimulates adiponectin mRNA expression in adipocytes.^43^ Thus, greater local concentrations of osteocalcin in BM might disproportionately increase adiponectin expression in MAT. Finally, differential exposure to other secreted factors that inhibit adiponectin expression and secretion, including glucocorticoids and pro-inflammatory cytokines,^38,44,45^ might also explain why production of adiponectin is greater in MAT than in WAT.

**Figure 3.**
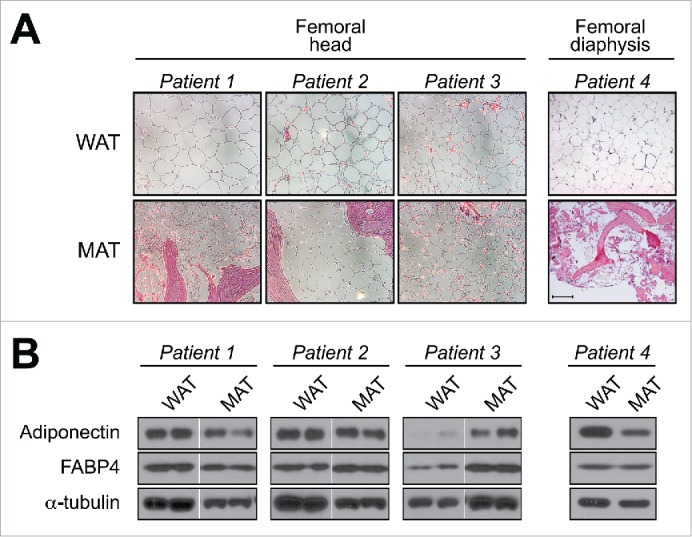
**Adiponectin expression in human femoral MAT**. Subcutaneous WAT and MAT were isolated from the femoral heads of patients undergoing hip-replacement (Patients 1–3) or from the femoral diaphysis of an amputation patient (Patient 4). (**A**) Representative micrographs of H&E-stained tissue sections. Scale bar = 200 µm. (**B**) Total protein was isolated from scWAT and MAT of each patient and expression of the indicated proteins was assessed by immunoblotting; similar results were observed for tissue samples obtained from two other hip-replacement patients (data not shown). Expression of α-tubulin was analyzed as a loading control, although expression was sometimes variable between each tissue type. For patients 1–3, MAT and scWAT lysates were run on non-adjacent lanes of the same gel, and therefore intervening lanes have been removed for ease of comparison. Both the Institutional Review Boards of the University of Michigan and of the Veterans Affairs Hospital of Ann Arbor, MI, approved the study involving hip-replacement patients (IRB number: HUM00053722). The University of Michigan Medical School Institutional Review Boards approved the study involving lower-limb amputation patients (IRB number: HUM00060733). Methods for histology and immunoblotting are as described previously.^4^

In addition to analyzing mouse and rabbit samples, we further characterized tibial MAT and subcutaneous WAT (scWAT) from humans. We found that tibial MAT expresses adiponectin protein at higher levels than scWAT, at least in the 3 patients studied.^4^ Together with our above results in mice and rabbits, our observations suggest that adiponectin transcripts and protein are expressed in MAT at levels similar to or greater than in WAT. However, this is at odds with two recent microarray studies by Liu *et al* and Poloni *et al*, in which adipocytes isolated from BM of mice or humans were found to have lower expression of adiponectin mRNA than adipocytes isolated from WAT (Fig. 2).^36,46^ This discrepancy could relate to the fact that we analyzed intact MAT while Liu *et al* and Poloni *et al* studied isolated adipocytes. Given that MAT and WAT do not consist exclusively of adipocytes, it is possible that non-adipocyte populations contribute to adiponectin expression in whole MAT. For example, osteoblasts reportedly express adiponectin transcripts and protein,^27,28,47^ and our human MAT samples and mouse caudal vertebrae clearly contained some ossified tissue (Fig. 2).^4^ However, osteoblasts express adiponectin mRNA at only 0.01% of adipocyte levels,^47^ whereas we found that rabbit tibial MAT expresses adiponectin mRNA at levels similar or greater than WAT, despite containing no trabecular bone.^4^ Moreover, our observations in mice show that adiponectin protein expression in caudal vertebrae is far greater than that in lumbar vertebrae, despite these tissues having similar bone content.^4^ Thus, we believe it unlikely that osteoblasts make any meaningful contribution to adiponectin expression in caudal vertebrae of mice or our human MAT samples. A second possibility relates to the fact that Liu *et al* and Poloni *et al* isolated adipocytes via collagenase treatment, a method that can alter cellular transcriptional profiles.^48^ Finally, and perhaps most intriguingly, is the possibility that adiponectin expression in MAT varies across different skeletal sites. Indeed, we recently revealed that properties of BM adipocytes are region-specific, such that MAT can be classified into two broad sub-types: regulated MAT (rMAT) exists in more proximal skeletal sites and consists of adipocytes interspersed with haematopoietic BM, while constitutive MAT (cMAT) exists in more distal regions (e.g., distal tibia, caudal vertebrae) and appears histologically similar to WAT, with few visible haematopoietic cells.^49^ These MAT subtypes also differ in their lipid composition and response to external stimuli.^49^ Therefore, it is notable that for our adiponectin studies we analyzed more cMAT-like tissue (i.e., from tails or distal tibiae), whereas Liu *et al* and Poloni *et al* studied adipocytes from regions of rMAT (i.e. pooled from femurs/tibiae, or from the iliac crest). We have since begun to investigate adiponectin expression in adipocytes isolated from rMAT, cMAT, and WAT of rodent models, and these studies are ongoing. We have also started to analyze adiponectin protein expression in more rMAT-like samples from human femurs, finding that adiponectin is expressed in these regions; however, unlike in tibial MAT, such expression is not always higher than that in scWAT (Fig. 3). This supports the possibility that adiponectin expression is higher in cMAT than in rMAT. Whether these MAT subtypes differ in adiponectin expression, or indeed other endocrine properties, clearly warrants further study (Figs. 2 and 5).

### Adiponectin secretion from MAT vs WAT

Despite these inconsistencies, it is clear that BM adipocytes do express adiponectin mRNA and protein; but what about adiponectin secretion? In this case the literature is less revealing. One earlier study noted adiponectin secretion from adipocytes differentiated *ex vivo* from human BM,^50^ while two more-recent papers confirm that adiponectin is secreted from primary adipocytes isolated from human femurs.^32,51^ To build on these observations, we analyzed adiponectin secretion during *ex vivo* culture of tibial MAT and WAT explants obtained from rabbits or humans.^4^ In both species, secretion of adiponectin was markedly higher from tibial MAT than from WAT, even after accounting for potential differences in total protein secretion and explant breakdown (Fig. 2).^4^ For the human samples, further analysis of LMW, MMW, and HMW adiponectin showed that each of these multimeric forms is also secreted more highly from MAT than from WAT (Fig. 2). While this finding is striking, the mechanistic basis remains to be determined. Adiponectin secretion is regulated by numerous factors, including PPARγ, SIRT1, the endoplasmic reticulum chaperones Ero1-Lα and ERp44, the enzyme DsbA-L, and the GTPase regulator FIP1,^52-55^ as well as the multi-ligand receptor sortilin, which directs adiponectin toward lysosomal degradation;^56^ hence, altered expression and/or activity of these factors could account for increased adiponectin secretion from cMAT. Another possibility relates to the impact of fatty acids on adiponectin production. Specifically, we recently discovered that cMAT has a lower proportion of saturated fatty acids than rMAT or WAT.^49^ Given that saturated fatty acids such as palmitate can suppress adiponectin transcript expression and protein secretion,^56^ decreased exposure to saturated fatty acids might lead to increased expression and secretion of adiponectin from cMAT. Future studies must explore these possibilities and also move beyond explant studies, which can adversely affect adipose tissue biology.^57^ Alternative approaches, such as analysis of arteriovenous differences in adiponectin concentrations across BM and WAT depots,^58^ could be one approach to determine the relative production of adiponectin by WAT and MAT *in vivo* (Fig. 2).

## Beyond the basics: MAT as a source of adiponectin during CR

### Supporting evidence

**Figure 4.**
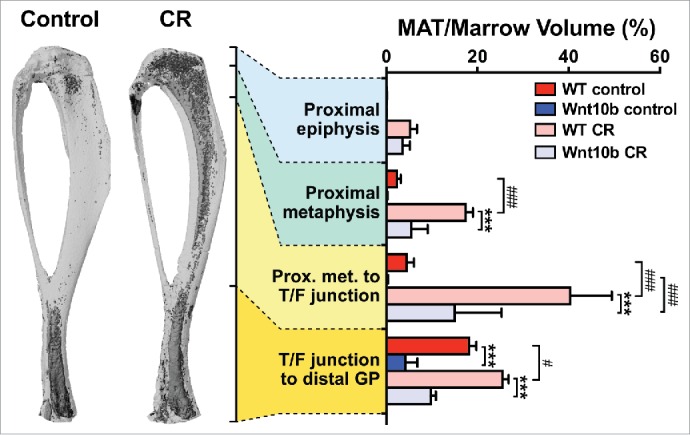
**Ocn-Wnt10b mice resist MAT expansion during CR**. To determine if Ocn-Wnt10b mice resist MAT formation, we stained tibiae with osmium tetroxide and analyzed MAT volume *in situ* by micro-CT scanning, as described previously.^4^ Representative images of osmium-stained tibiae from wild-type mice, fed a control or CR diet, are shown on the left of the figure; osmium-stained MAT appears as darker regions within the bones. MAT volume was then quantified for each of the indicated tibial regions and normalized to total marrow volume to give percentage of MAT volume, as shown in the graph on the right. Data in the graph are presented as mean +/− standard deviation of the following numbers of mice: WT control, n = 6; Wnt10b control, n = 4; WT CR, n = 5; and Wnt10b CR, n = 6. For each diet group, significant differences between WT and Wnt10b mice are indicated by *** (*P* < 0.001). Within each genotype, significant differences between control and CR diets are indicated by # (*P* < 0.05) or ### (*P* <0.001).

Having confirmed that MAT expresses and secretes adiponectin, we next sought to determine if MAT contributes to circulating adiponectin. An ideal tool for addressing this question would be an animal model that lacks MAT but not WAT, or vice versa. Unfortunately, such a model has yet to be firmly established; however, we were extremely fortunate in that our lab had previously developed Ocn-Wnt10b mice, which express the secreted ligand Wnt10b in osteoblasts. Wnt10b promotes osteoblastogenesis, and therefore these mice have increased bone mass.^59^ Wnt10b also inhibits adipogenesis,^60^ leading us to speculate that Ocn-Wnt10b mice might also lack MAT. Our initial analyses of proximal tibiae, from the proximal metaphysis to the tibia-fibula junction, showed that the while Ocn-Wnt10b mice tend to have less MAT than their control littermates, this difference does not reach statistical significance.^4^ However, additional experiments revealed that Ocn-Wnt10b mice significantly resist expansion of this MAT depot during CR.^4^ We have since extended these analyses to whole tibiae (Fig. 4), revealing that MAT is also decreased in the proximal metaphysis and distal tibia of CR-fed Ocn-Wnt10b mice and that, even without CR, Ocn-Wnt10b mice have significantly less distal tibial MAT than their control littermates (Fig. 4). These new data also highlight that CR-associated MAT expansion occurs predominantly in the proximal tibia rather than the distal tibia. This further supports the designation of these sites as rMAT and cMAT, respectively.

These observations demonstrate that Ocn-Wnt10b mice have moderately decreased MAT volume without CR and robustly resist MAT expansion during CR. What are the consequences for circulating adiponectin? We found no differences in control-fed mice but, strikingly, CR-associated hyperadiponectinemia is significantly blunted in the Ocn-Wnt10b mice.^4^ Importantly, this occurs despite unaltered expression of adiponectin transcripts or protein in WAT.^4^ It remains possible that genotype-dependent differences in adiponectin half-life or secretion from WAT contribute to this striking phenotype; however, we found that WAT expression of ERp44 and Dsb-AL, key regulators of adiponectin secretion, is unaltered by diet or genotype.^4^ Thus, Ocn-Wnt10b mice likely resist CR-associated hyperadiponectinemia because of impaired MAT expansion, rather than altered adiponectin production from WAT.^4^ Together these observations provide direct evidence that MAT expansion is required for hyperadiponectinemia during CR.

Other recent studies further support this conclusion. For example, Zgheib *et al* investigated the effects of separation-based anorexia (SBA), a unique model of CR in mice. They find that SBA causes many typical effects of CR, such as weight loss and hypoleptinemia; however, it does not lead to hyperadiponectinemia.^21^ More recent research from this group shows that MAT expansion also fails to occur during SBA (Christophe Chauveau, personal communication). Similarly, we recently revealed that CR in rabbits leads to decreased body mass, WAT mass, and circulating leptin, but without hyperadiponectinemia or MAT expansion.^61^ Thus, data from three distinct animal models suggest that MAT expansion is necessary for hyperadiponectinemia during CR, supporting the conclusion that MAT contributes to increased circulating adiponectin in this context.

### Functional consequences

Adiponectin acts on numerous target tissues and cell types to exert diverse systemic effects on metabolic homeostasis, vascular function, inflammation, and other systems. What, then, are the consequences of MAT's contribution to hyperadiponectinemia during CR? Our studies in Ocn-Wnt10b mice reveal that, during CR, these mice not only resist hyperadiponectinemia but also have altered adaptations in skeletal muscle, including decreased expression of transcripts related to mitochondrial function and increased activity of AMP-activated protein kinase.^4^ Beyond skeletal muscle, other responses to CR were similar between Ocn-Wnt10b and control mice, including enhanced glucose tolerance and altered hepatic transcription.^4^ From this one could infer that the metabolic impact of MAT expansion and/or hyperadiponectinemia during CR is limited to skeletal muscle; however, there are several important caveats. Firstly, there are many other metabolic effects of CR that we did not assess, including altered energy expenditure, substrate utilization, and β-cell function, to name but a few. Secondly, while CR-associated MAT expansion and hyperadiponectinemia are blunted in the Ocn-Wnt10b mice, these effects still occur. Thus, we are currently pursuing additional approaches to determine more comprehensively the consequences of CR-associated MAT expansion.

### Additional considerations

**Figure 5.**
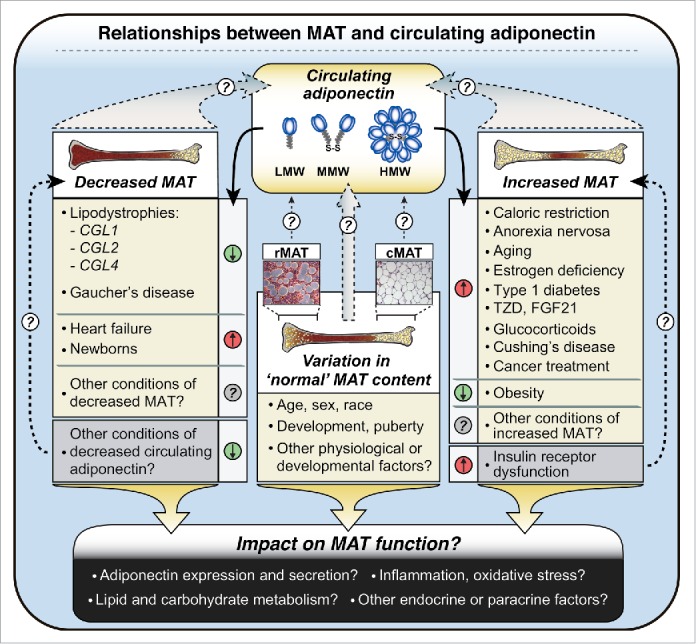
**Potential relationships between MAT and circulating adiponectin in health and disease**. Circulating adiponectin is represented as low-molecular-weight (LMW) trimers, middle-molecular-weight (MMW) hexamers, and high-molecular-weight (HMW) dodecamers, although HMW forms may consist of even larger multimers. MAT content varies in ‘normal’ physiological and developmental contexts, with further decreases or increases occurring in adverse or pathological conditions, as indicated. In some cases decreases or increases in MAT are paralleled by similar changes in circulating adiponectin (e.g. decreases in CGL1, CGL2, CGL4; increases in CR, AN), while in other conditions MAT and circulating adiponectin change in opposite directions (e.g., heart failure; obesity). Future studies must address the relative contributions of rMAT and cMAT (represented here by micrographs of rMAT and cMAT from rabbits), as well as how these diverse physiological and clinical conditions impact not just MAT content, but also MAT function.

Although there is much supporting evidence, it is important to consider any observations that question the contribution of MAT to hyperadiponectinemia during CR. For example, several papers report that CR does increase adiponectin mRNA and/or protein expression in WAT (Table 1), suggesting that WAT does contribute to hyperadiponectinemia under these conditions of CR. Surprisingly, one of these studies also finds that CR leads to increased adiponectin expression in skeletal muscle, with adiponectin protein expression being higher in skeletal muscle than in visceral WAT.^15^ From these surprising observations, the authors propose that skeletal muscle may contribute to hyperadiponectinemia during CR. Finally, in subjects with AN, both MAT and circulating adiponectin inversely correlate with bone mineral density,^25^ and therefore one might expect BM adiposity to positively associate with circulating adiponectin in AN subjects. However, in our recent analysis of MAT and adiponectin in control and AN subjects, there was no significant association between MAT and circulating adiponectin (P. Fazeli and A. Klibanski, unpublished observations). One limitation is that these MRI-based measurements focused only on vertebral and femoral MAT,^4^ which might hold less influence on circulating adiponectin than the more cMAT-like adipocytes within tibial MAT (Fig. 3). Moreover, BM adiposity alone might not sufficiently reflect the contribution of MAT to CR-associated hyperadiponectinemia. Indeed, it seems plausible that CR or AN could alter adiponectin production from MAT, thereby impacting circulating levels without alterations in total MAT mass (Figs. 2 and 5). However, while such knowledge is limiting in the case of WAT (Table 1), for MAT it is entirely lacking. Future research must address these questions, in particular how CR or AN impacts adiponectin secretion from these and other tissues.

## Does MAT contribute to circulating adiponectin in other contexts?

### Lessons from lipodystrophies

Our studies in Ocn-Wnt10b mice provide the most compelling evidence that MAT contributes to hyperadiponectinemia during CR, because of the unique ability of these mice to resist the formation of MAT but not WAT. Further insights could be gleaned from the converse phenotype, i.e., a lack of WAT but not MAT. Unfortunately, animal models with this phenotype are lacking. However, differential loss of WAT and MAT can occur in human patients with lipodystrophies, acquired or inherited conditions characterized by the impaired formation, progressive loss, or redistribution of adipose tissue.^62^ Consequently, WAT is either partially or totally absent; however, MAT is sometimes maintained. Therefore, these conditions might provide further insights into the contributions of WAT and MAT to circulating adiponectin. For example, is circulating adiponectin greater in lipodystrophic subjects whose MAT is preserved, compared to those who lack MAT? As shown in Table 2, this is sometimes, but not always, the case. Thus, subjects with acquired partial lipodystrophy (APL) have partial loss of visceral and subcutaneous WAT, while MAT is preserved and adiponectin concentrations are normal. A similar situation exists for familial partial lipodystrophy (FPLD), although this can feature increased visceral WAT; slight decreases in circulating adiponectin were also reported in one group of FPLD subjects (Fig. 2). Conversely, in other classes of lipodystrophies both MAT and circulating adiponectin are decreased. These include the partial lipodystrophy associated with antiretroviral therapy for HIV, as well as congenital generalized lipodystrophies (CGL) caused by mutations in *AGPAT2* (CGL1) or *BSCL2* (CGL2). For CGL4, caused by *PTRF* mutations, MAT was detected in MRI scans of one patient,^63^ but for most CGL4 patients MAT content has not been reported. However, we recently revealed that lack of *Ptrf* in mice is associated with loss of rMAT but not cMAT,^49^ suggesting partial loss of MAT in CGL4 (Table 2). Together, these observations support the possibility that preservation of MAT is necessary if normal circulating adiponectin levels are to be maintained. However, in patients with acquired generalized lipodystrophy (AGL) and in the only known CGL3 subject, circulating adiponectin is decreased despite the presence of MAT (Table 2). Thus, the presence of MAT does not guarantee maintenance of normal circulating adiponectin concentrations.

While such observations can be informative, their interpretation is difficult because lipodystrophies are typically associated with metabolic dysregulation, such as insulin resistance and dyslipidemia, which themselves adversely affect circulating adiponectin concentrations. Moreover, analysis of MAT content in lipodsytrophic patients has been extremely limited, while essentially nothing is known about how the underlying clinical defects impact adiponectin production by MAT.

Mouse models of lipodystrophy are similarly limited, but can yield useful insights into the relationship between WAT, MAT, and adiponectin. In one notable study, Colombo *et al* investigated the impact of WAT transplantation in A-ZIP/F-1 mice, a well-established mouse model of lipodystrophy. In these mice WAT is absent; circulating levels of adiponectin are decreased by 98%; and those of leptin, another adipokine, are over 99% lower than in controls.^64^ Upon transplanting wild-type scWAT into A-ZIP/F-1 mice, circulating leptin increased to 40% of wild-type concentrations, while circulating adiponectin concentrations reached only 4% of those in wild-type controls.^64^ The authors state, *“The serum levels of adiponectin achieved by WAT transplantation were very low. It is not clear why adipose tissue transplantation to a level ∼25% of wild-type WAT weight and producing nearly wild-type levels of leptin gave adiponectin levels only ∼4% of wild-type.”* This suggests that, at least in this context, WAT makes only a minor contribution to circulating adiponectin. Although they would have been unaware at the time, it has since been confirmed that A-ZIP/F-1 mice also lack MAT.^25^ Might this explain why WAT transplantation alone has only a negligible effect on circulating adiponectin?

Another recently published mouse model may provide further important insights. In an elegant approach, the Scherer lab generated mice in which expression of C/EBPα in mature adipocytes can be inducibly ablated via treatment with doxycycline.^42^ Development of gWAT is postnatal, whereas that of scWAT occurs during embryogenesis.^65^ Thus, perinatal treatment of these mice with doxycycline, before substantial gWAT development, leads to loss of C/EBPα in scWAT but not gWAT.^42^ While this does not affect scWAT mass, it is associated with a 34% decrease in circulating adiponectin concentrations, leading the authors to conclude that scWAT “*contributes about one-third of the adiponectin in systemic circulation.”* However, it is notable that the MAT phenotype of these mice was not investigated. Indeed, we recently demonstrated that cMAT adipocytes have even greater expression of C/EBPα than adipocytes in scWAT,^49^ which suggests that cMAT might be even more susceptible than scWAT to C/EBPα deletion. Therefore, in the Scherer lab's unique mouse model it seems highly plausible that C/EBPα ablation would impair adiponectin production from MAT, thereby leading to decreased circulating adiponectin. We eagerly await studies addressing this possibility.

### Insulin receptor dysfunction and hyperadiponectinemia

The above studies underscore the potential of monogenic human diseases and transgenic mouse models to clarify our knowledge of MAT's contribution to circulating adiponectin. This applies not only to conditions of decreased circulating adiponectin, but also to states characterized by hyperadiponectinemia. In particular, marked increases in circulating adiponectin occur in patients with insulin receptoropathies caused by insulin receptor antibodies or mutations in the insulin receptor.^66,67^ Hyperadiponectinemia also occurs in FIRKO mice, which lack the insulin receptor in adipose tissue.^68^ Thus, insulin receptor dysfunction is associated with increased circulating adiponectin. While the underlying reasons remain unclear, several observations support the possibility that MAT plays a role. For example, insulin directly suppresses adiponectin expression in human MAT,^32^ suggesting that insulin receptor dysfunction might increase MAT adiponectin production. Moreover, MAT expansion occurs in conditions of hypoinsulinemia, such as CR and type 1 diabetes,^25^ which suggests that insulin might suppress MAT formation. Thus, there is clear rationale for investigating if MAT is increased in FIRKO mice or in humans with insulin receptoropathies, as this might explain the idiopathic hyperadiponectinemia that occurs in these conditions (Fig. 5).

### Associations between MAT and adiponectin: not restricted to caloric restriction

Our studies to date have focused on the contribution of MAT and WAT to hyperadiponectinemia during CR. However, increases in both MAT and circulating adiponectin also occur in many other diverse conditions, including aging, estrogen deficiency, type I diabetes, and in response to pharmacological agents such as TZDs, glucocorticoids and fibroblast growth factor-21 (FGF21) (Fig. 5).^8,25,36,69-73^ Conversely, both MAT and circulating adiponectin are decreased in Gaucher's disease.^74-76^ This suggests that loss of MAT may lead to hypoadiponectinemia, consistent with our above discussion of lipodystrophies (Table 2). Whether these conditions also feature a significant positive correlation between MAT and circulating adiponectin has yet to be established; however, such an association has been noted in patients with Cushing's Disease.^77^ Finally, our recent work further reveals that both MAT and circulating adiponectin increase in patients undergoing chemotherapy or radiotherapy for cancer.^4^ Based on these observations, it is tempting to speculate that MAT influences circulating adiponectin levels in states other than CR (Fig. 5). This relationship might also extend beyond adverse clinical conditions. Indeed, ethnic differences in healthy adults have been reported for both circulating adiponectin and BM adiposity, with each of these being higher in Caucasians than in adults of African origin.^78,79^ Moreover, a positive correlation between MAT volume and serum adiponectin was recently reported in healthy Caucasian girls.^80^

The above evidence suggests that MAT might contribute to circulating adiponectin beyond CR, both in clinical contexts and in healthy populations (Fig. 5). However, it must be emphasized that, in other conditions, there is discordance between MAT content and circulating adiponectin. For example, heart failure is associated with hyperadiponectinemia, despite MAT loss.^81,82^ Similarly, circulating adiponectin concentrations in newborn humans are 2- to 3-fold higher than in healthy adults,^83^ even though MAT is essentially absent in newborns.^81^ Finally, in adult humans it is well established that circulating adiponectin levels are higher in females than in males;^5^ however, most studies to date suggest that males have more MAT than females.^25^ Thus, while increases in MAT are paralleled by increased circulating adiponectin in many conditions, this relationship is not universal (Fig. 5).

### MAT and adiponectin in obesity

This discordance is perhaps most notable in the case of obesity and insulin resistance. When we began to study the relationship between MAT and circulating adiponectin it had not yet been established if MAT volume was altered in such adverse metabolic conditions; however, more recent studies report increased MAT in high-fat-diet-fed mice^84-86^ and in humans with visceral obesity and dyslipidemia or type 2 diabetes.^87-89^ Unfortunately none of these studies assessed circulating adiponectin, and therefore it remains unclear if this is related to MAT content in such conditions. The clinical studies were also limited to small groups of subjects and therefore await verification in larger cohorts. Nevertheless, these findings raise the question: if MAT truly is a source of adiponectin, then why does circulating adiponectin decrease in obese, insulin-resistant states, when MAT increases?

Although this question might quickly spring to mind, by this logic one would also question if WAT is a source of adiponectin; after all, obesity is defined by excessive WAT accumulation, yet this has not cast doubt on the contribution of WAT to circulating adiponectin. Of course, it is now well established that obesity and insulin resistance lead to WAT dysfunction, including excessive inflammation and oxidative stress, which impairs adiponectin production from WAT. Increasing evidence demonstrates that obesity and insulin resistance also promote oxidative stress and inflammation within BM.^90-92^ Adipocytes in MAT might be particularly susceptible to such stress, given their relatively high expression of proinflammatory genes.^36^ Thus, it seems likely that obesity would also lead to adipocyte dysfunction within MAT, thereby compromising production of adiponectin. Determining how obesity impacts MAT function must therefore be a priority of future research (Figs. 2 and 5).

## Concluding Perspectives

Our research to date provides compelling evidence that MAT contributes to increased circulating adiponectin during CR. The basis of this phenomenon might be further clarified by studies beyond MAT, including more widespread analysis of adiponectin secretion from WAT and its clearance from the circulation, each of which has been largely ignored (Table 1). However, given the limited study of MAT (Fig. 1), it is not surprising that many of the key questions are focused on this tissue. For example, to what extent does adiponectin expression and secretion vary between rMAT, cMAT, and WAT, and what are the underlying mechanisms (Fig. 2)? Moreover, are these characteristics affected by CR, obesity, aging, lipodystrophies, or other physiological or pathological conditions in which MAT and/or circulating adiponectin is altered (Figs. 2 and 5)? Similarly, to what degree does the relationship between MAT and circulating adiponectin extend to other physiological or pathological conditions (Fig. 5)?

The function of MAT as an endocrine organ could have enormous implications. Our studies in Ocn-Wnt10b mice suggest that MAT expansion contributes to CR-associated adaptations in skeletal muscle,^4^ demonstrating that MAT can exert systemic effects. However, whether this is through adiponectin or other endocrine factors remains to be determined, as does the full extent of MAT's systemic actions during CR. The endocrine impact of MAT in other contexts, beyond CR, also demands further investigation.

While it remains possible that MAT produces other endocrine factors, thus far we have focused on adiponectin. Here, a major point of interest would be to determine if MAT influences circulating adiponectin in healthy humans. As mentioned above, one study reports a positive relationship between MAT and adiponectin in healthy girls, albeit in a rather limited context.^80^ Several genome-wide association studies have identified genetic variants that influence circulating adiponectin,^93-95^ and therefore it would be informative to investigate if these variants also impact MAT formation or function. Indeed, huge insights into MAT biology, beyond its relationship to adiponectin, might be gleaned from more widespread analysis of MAT across larger human populations. Achieving this goal would not be straightforward. For example, determination of MAT content non-invasively relies on MRI, which is expensive and time consuming, making large-scale studies difficult. Even if this were achievable, would such measurements of MAT content alone be sufficient to inform us about the contribution of MAT to circulating adiponectin, or indeed to other physiological or pathological phenomena? As for WAT in obesity, alterations in MAT function, perhaps undetectable by MRI, might have a far greater impact than changes in MAT content alone (Fig. 5). This underscores the need to more directly analyze MAT function though both preclinical and clinical studies, including establishing the global characteristics of MAT in comparison to other adipose depots.

Clearly, there is much work to be done; however, interest in MAT formation and function is growing and research in this field is gathering momentum. The future therefore holds great promise for further expanding our understanding of MAT, not only as a source of adiponectin, but also more broadly in the context of health and disease.

## Abbreviations

AGL: acquired generalized lipodystrophy
AN: anorexia nervosa
APL: acquired partial lipodystrophy
BAT: brown adipose tissue
BM: bone marrow
C/EBPα: CCAAT/enhancer-binding protein-α;
CGL: congenital generalized lipodystrophy
cMAT: constitutive MAT
CR: caloric restriction
FABP4: fatty-acid-binding protein 4
FGF21,: fibroblast growth factor-21
FPLD,: familial partial lipodystrophy
HMW: High-molecular-weight adiponectin
HSL: Hormone-sensitive lipase
LMW,: Low-molecular-weight adiponectin
MAT: marrow adipose tissue
MMW: Middle-molecular-weight adiponectin
PPARγ,: peroxisome proliferator-activated receptor-γ;
rMAT: regulated MAT
SBA: separation-based anorexia
TZD: thiazolidinedione
WAT: white adipose tissue
iWAT: inguinal WAT
gWAT: gonadal WAT
pWAT: perirenal WAT
